# Pre‐clinical evidence of a dual NADPH oxidase 1/4 inhibitor (setanaxib) in liver, kidney and lung fibrosis

**DOI:** 10.1111/jcmm.17649

**Published:** 2023-01-19

**Authors:** Victor J. Thannickal, Karin Jandeleit‐Dahm, Cédric Szyndralewiez, Natalie J. Török

**Affiliations:** ^1^ John W. Deming Department of Medicine Tulane University School of Medicine New Orleans Louisiana USA; ^2^ Southeast Louisiana Veterans Healthcare System New Orleans Louisiana USA; ^3^ Department of Diabetes, Central Clinical School Monash University Melbourne Victoria Australia; ^4^ Calliditas Therapeutics Suisse SA Geneva Switzerland; ^5^ Division of Gastroenterology and Hepatology, Department of Medicine Stanford University Stanford California USA; ^6^ Present address: Pherecydes Pharma Nantes France

**Keywords:** fibrosis, kidney diseases, liver cirrhosis, liver diseases, NADPH oxidases, pulmonary fibrosis, reactive oxygen species, setanaxib

## Abstract

Fibrosis describes a dysregulated tissue remodelling response to persistent cellular injury and is the final pathological consequence of many chronic diseases that affect the liver, kidney and lung. Nicotinamide adenine dinucleotide phosphate (NADPH)‐oxidase (NOX) enzymes produce reactive oxygen species (ROS) as their primary function. ROS derived from NOX1 and NOX4 are key mediators of liver, kidney and lung fibrosis. Setanaxib (GKT137831) is a first‐in‐class, dual inhibitor of NOX1/4 and is the first NOX inhibitor to progress to clinical trial investigation. The anti‐fibrotic effects of setanaxib in liver, kidney and lung fibrosis are supported by multiple lines of pre‐clinical evidence. However, despite advances in our understanding, the precise roles of NOX1/4 in fibrosis require further investigation. Additionally, there is a translational gap between the pre‐clinical observations of setanaxib to date and the applicability of these to human patients within a clinical setting. This narrative review critically examines the role of NOX1/4 in liver, kidney and lung fibrosis, alongside the available evidence investigating setanaxib as a therapeutic agent in pre‐clinical models of disease. We discuss the potential clinical translatability of this pre‐clinical evidence, which provides rationale to explore NOX1/4 inhibition by setanaxib across various fibrotic pathologies in clinical trials involving human patients.

## 1 INTRODUCTION

Fibrosis describes the pathological wound‐healing response to injury that leads to healthy tissue becoming displaced by permanent scar tissue and is the final pathological consequence of many chronic inflammatory diseases.^1^, ^2^ Damage‐induced fibrosis can be caused by different stimuli, including autoimmune conditions, persistent infections, tissue injury, chemical damage and radiation.^1^

Fibrosis is characterized by the deposition of extracellular matrix (ECM) material, such as collagen and fibronectin, and contributes to tissue repair in all organs.^3^, ^4^ Upon injury, local fibroblasts become activated and differentiate into contractile cells known as myofibroblasts, which synthesize ECM components and, together with a modest inflammatory response, initiate the wound‐healing process.^3^, ^4^ When tissue damage is minor or non‐persistent, limited deposition of ECM components occurs, which are quickly eliminated to restore normal tissue architecture and preserve tissue function.^3^ However, severe or persistent tissue damage can lead to fibrosis due to chronic inflammation and dysregulated repair.^1^, ^2^, ^3^, ^4^ Here, tissue remodelling and repair processes occur simultaneously, characterized by excessive ECM deposition and the formation of permanent scar tissue.^1^, ^2^, ^3^, ^4^ As such, fibrosis can lead to distorted tissue architecture and impaired organ function.^1^, ^2^, ^3^ In some diseases, such as liver cirrhosis, diabetic nephropathy and idiopathic pulmonary fibrosis (IPF), extensive fibrosis‐induced tissue remodelling can lead to organ failure and death.^1^, ^2^, ^3^, ^4^

The onset and progression of fibrosis is orchestrated by many profibrotic metabolites, including reactive oxygen species (ROS).^5^ ROS are highly reactive oxygen‐derived molecules encompassing free radicals (e.g., superoxide anion [O_2_^•‐^]), and non‐radical species (e.g., hydrogen peroxide [H_2_O_2_]).^6^, ^7^ Oxidative stress denotes the state in which ROS levels exceed the protective capacity of cellular antioxidant defence systems.^7^ Whilst controlled generation of ROS is important for normal physiological processes, such as cellular signalling and antimicrobial immunity, excessive ROS production can contribute to pathophysiological consequences, including fibrosis development and persistence.^5^, ^7^, ^8^ Specifically, ROS activate and mediate the effects of profibrotic cytokines, namely transforming growth factor‐beta (TGF‐β).^5^, ^9^ In turn, TGF‐β triggers ROS production and suppresses cellular antioxidant levels, which induces oxidative stress and contributes to fibrosis progression.^5^, ^9^

ROS can be generated as by‐products of the mitochondrial electron transport chain.^5^ Specific enzymes can also produce ROS, including xanthine oxidase, cytochrome P450 oxidases, cyclooxygenases, lipoxygenases and nicotinamide adenine dinucleotide phosphate (NADPH)‐oxidases (NOX).^5^, ^8^ The majority of these enzymes generate ROS as by‐products of their enzymatic activities, whereas NOX enzymes produce ROS as their primary catalytic end‐product, either as O_2_^•‐^ or H_2_O_2_ via a NADPH‐dependent reduction of molecular oxygen.^8^, ^10^, ^11^

The NOX enzyme family constitutes seven members: NOX1–5 and dual oxidases (DUOX) 1 and 2.^10^, ^11^ The NOX isoforms differ in how they are regulated, their subcellular localization and the type of ROS produced (Table 1).^11^, ^12^ Recent evidence has demonstrated the importance of NOX enzymes in the development of tissue inflammation and fibrosis.^10^ NOX1 and NOX4 have been shown to drive fibrotic pathologies in various organs, including the liver,^13^, ^14^, ^15^, ^16^, ^17^, ^18^ kidney^19^, ^20^, ^21^, ^22^, ^23^, ^24^, ^25^ and lung.^26^, ^27^, ^28^, ^29^ Thus, the pharmacological inhibition of NOX1/4 offers a potentially promising therapeutic intervention for a range of fibrotic pathologies, particularly those relating to the liver, kidney and lung.

**TABLE 1:** Tissue distribution and regulation of NOX/DUOX isoforms.

Enzyme	Site of expression	Known regulatory factors	ROS produced
NOX1	Inducible; colon, vascular smooth muscle, endothelium, placenta, prostate, uterus, skin, osteoclasts, retinal pericytes	p22^phox^, NOXO1, NOXA1, Rac1	Superoxide
NOX2	Phagocytes, β lymphocytes, cardiomyocytes, hepatocytes, vascular smooth muscle, fibroblasts, skeletal muscle, neurons, lung, carotid body, kidney	p22^phox^, p47^phox^, p67^phox^, Rac1, Rac2	Superoxide
NOX3	Foetal kidney, inner ear, neurons	p22^phox^, NOXO1, Rac1	Superoxide
NOX4	Kidney, vascular smooth muscle, endothelium, osteoclasts, fibroblasts, keratinocytes, cardiomyocytes, bone, ovary, pancreas, eye, skeletal muscle, testes	p22^phox^ (constitutively active)	Superoxide/hydrogen peroxide
NOX5	Spleen, sperm, testes, ovary, prostate and cerebrum	Calcium and phosphorylation	Superoxide
DUOX1	Thyroid, cerebellum and lungs	Calcium and phosphorylation	Hydrogen peroxide
DUOX2	Thyroid, colon, pancreatic islets and prostate	Calcium and phosphorylation	Hydrogen peroxide

*Note*: Adapted from Teixera et al., 2016 and Lambeth 2004.^11^, ^12^

Abbreviations: DUOX, dual oxidase; NADPH, nicotinamide adenine dinucleotide phosphate; NOX, NADPH oxidase; NOXA1, NOX activator 1; NOXO1, NOX organizer 1.

**FIGURE 1:**
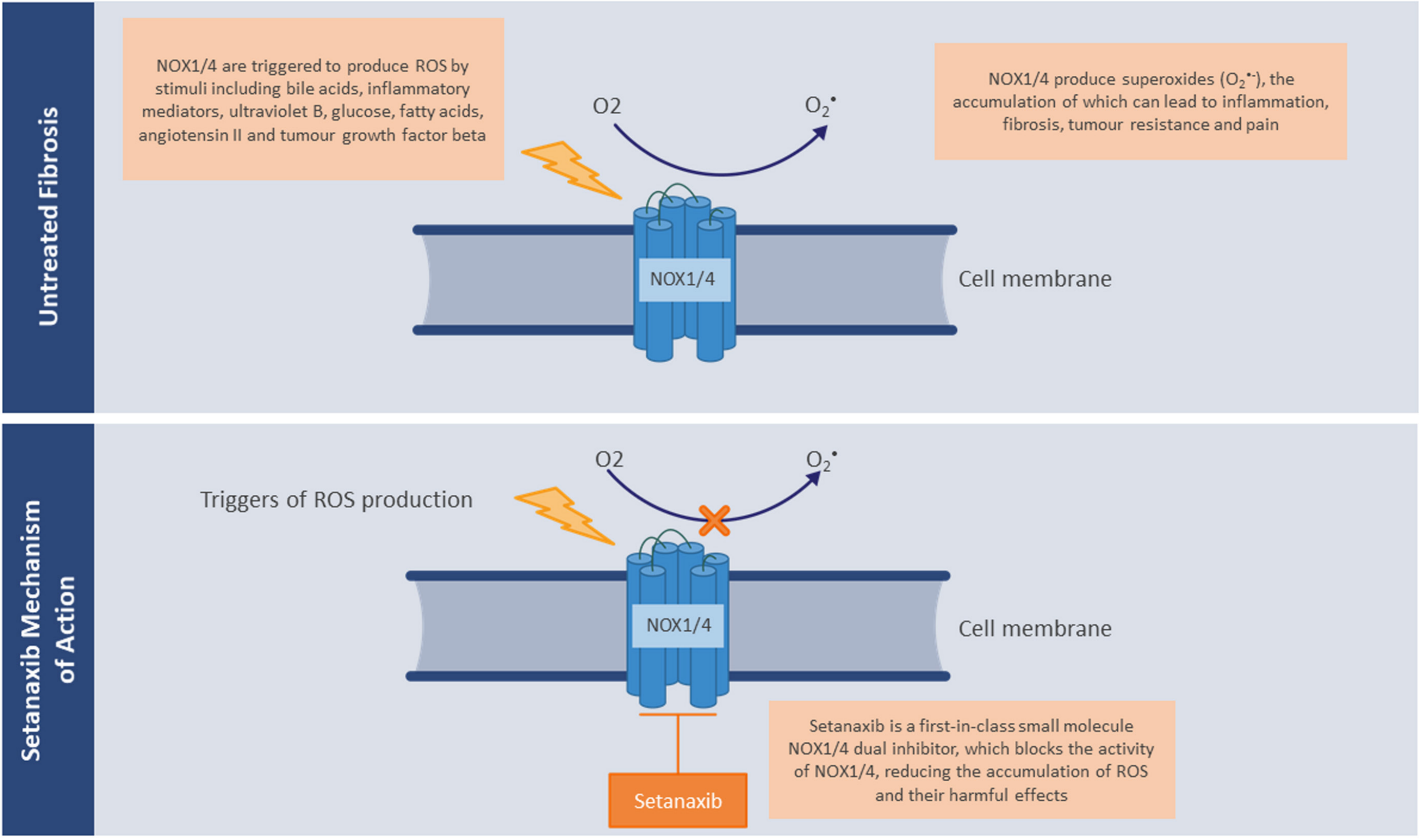
Setanaxib (GKT137831) mechanism of action. Adapted from Paik and Brenner, 2011 and Teixeira et al., 2017.^11^, ^30^ NOX, nicotinamide adenine dinucleotide phosphate (NADPH) oxidase; O_2_, molecular oxygen; O_2_^•^, superoxide anion; ROS, reactive oxygen species.

Setanaxib, formerly GKT137831, is a first‐in‐class NOX1/4 dual inhibitor that blocks the activity of NOX1/4, therefore reducing ROS production and concurrent harmful fibrotic effects (Figure 1).^11^, ^30^ Setanaxib is the first NOX inhibitor to progress through pre‐clinical testing to clinical development. To date, setanaxib has been investigated in patients with type 2 diabetes and albuminuria (phase 2; NCT02010242),^31^ and in patients with primary biliary cholangitis (PBC; phase 2; NCT03226067),^32^ with results from these studies yet to be published. Setanaxib is also currently undergoing clinical trial investigations for patients with PBC (phase 2b/3; NCT05014672),^33^, ^34^ IPF (phase 2; NCT03865927),^35^ type 1 diabetes‐related kidney disease (phase 2; ACTRN12617001187336),^36^, ^37^ and squamous cell carcinoma of head and neck (phase 2; NCT05323656).^38^

This narrative review critically discusses the role of NOX1/4‐mediated ROS production in liver, kidney and lung fibrosis, the pre‐clinical evidence in support of setanaxib as a therapeutic agent in the pre‐clinical setting, and the potential clinical translatability of such pre‐clinical evidence. This rationalizes the exploration of NOX1/4 inhibition by setanaxib across various fibrotic pathologies in clinical trials involving human patients.^33^, ^35^

## 2 LIVER FIBROSIS

### 2.1 Role of NOX1/4 in liver fibrosis

Liver fibrosis is caused by chronic exposure to hepatic injuries of diverse aetiology, including hepatitis B or C, autoimmune diseases such as PBC, alcoholic liver disease, non‐alcoholic fatty liver disease (NAFLD) and non‐alcoholic steatohepatitis (NASH), and is sequela of most chronic liver diseases.^10^, ^39^ Liver fibrosis is thought to result from the interaction between common fibrotic pathways linked to an uncontrolled tissue repair response and dysregulated fibrolytic pathways and is characterized by the remodelling of hepatic tissue with abundant ECM components and hepatocyte loss and apoptosis.^10^, ^11^, ^39^ As such, liver fibrosis distorts hepatic architecture and impairs liver function, which may result in portal hypertension, cirrhosis, liver failure and hepatocellular carcinoma.^11^, ^39^

The dysregulated liver repair process is thought to be initiated by epithelial injury, which is often associated with the release of danger‐associated molecular patterns (DAMPs) and pathogen‐associated molecular patterns (PAMPs).^10^ DAMPs and PAMPs are recognized by hepatic‐resident macrophages or recruited monocytic cells, triggering liver inflammation.^10^ Liver inflammation encompasses the recruitment and activation of immune cells by proinflammatory mediators secreted from injured hepatocytes, namely chemokines, tumour necrosis factor‐alpha (TNF‐α) and other mediators and their subsequent infiltration into liver tissue.^10^ Injured hepatocytes and activated immune cells also secrete profibrotic mediators, such as TGF‐β, which alongside proinflammatory mediators, activate resident hepatic stellate cells (HSCs) and trigger their transdifferentiation into myofibroblast‐like cells that have proinflammatory and profibrotic properties.^10^, ^39^

**FIGURE 2:**
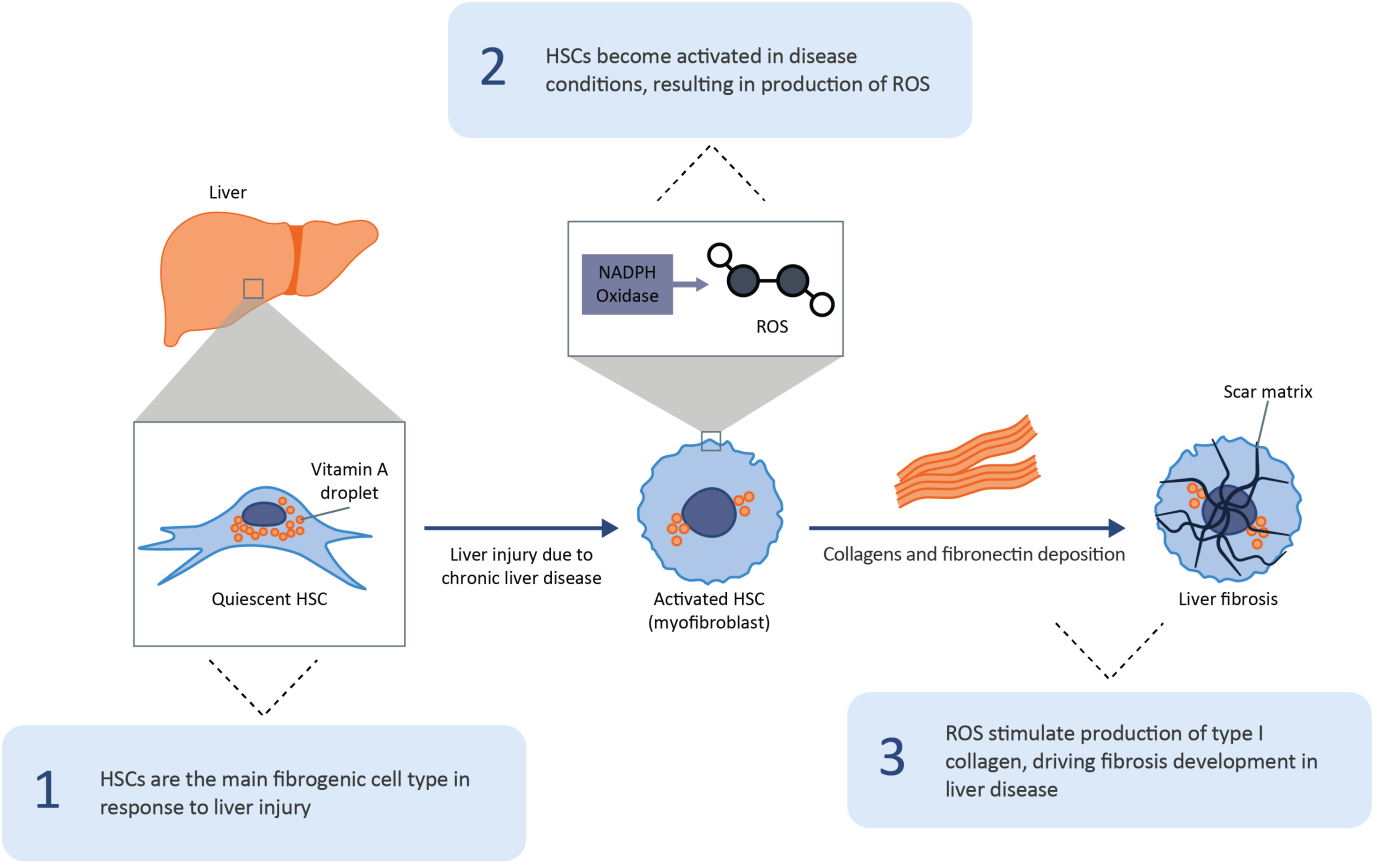
NOX1/4‐mediated ROS production in liver fibrosis. HSC, hepatic stellate cell; NADPH, nicotinamide adenine dinucleotide phosphate; NOX, NADPH oxidase; ROS, reactive oxygen species.

Oxidative stress is a key aetiological factor that initiates liver fibrosis. NOX1/4‐derived ROS, generated by hepatocellular injury, enhance the progression of liver fibrosis by stimulating type I collagen production and mediating the profibrotic effects of TGF‐β, including HSC activation and the continued production of proinflammatory cytokines and ECM components by myofibroblasts (Figure 2).^40^ NOX1 expression in HSCs becomes upregulated following both carbon tetrachloride (CCl_4_)‐ and bile duct ligation (BDL)‐induced liver fibrosis. This is consistent with observations of suppressed ROS generation, HSC activation and liver fibrosis in NOX1 knockout (KO) mice compared with wild‐type (WT) mice.^17^, ^41^, ^42^ Similarly, NOX4 expression was induced in HSCs isolated from mice after BDL, in hepatocytes isolated from CCl_4_‐treated mice and in mice subjected to fast‐food diet (FFD) and choline‐deficient amino acid‐defined (CDAA) dietary models of NASH.^14^, ^16^, ^43^ Importantly, ROS production, TGF‐β‐induced HSC activation and hepatocyte apoptosis were reduced in NOX4 KO mice compared with WT mice after CCl_4_ and BDL treatment, and following exposure to FFD and CDAA diets.^14^, ^16^, ^17^, ^43^

While there is evidence that NOX1/4 are important in progressing liver fibrosis, the cell‐type‐related functional differences of NOX1/4 in fibrotic pathologies must be considered. For example, NOX4 induces the activation and transdifferentiation of HSCs to myofibroblasts while triggering apoptotic cell death in hepatocytes, thus contributing to fibrosis development via both direct and indirect mechanisms.^16^, ^43^ In contrast, abundant NOX1 expression was demonstrated in liver sinusoidal endothelial cells (LSECs) isolated from mice fed a high‐fat and high‐cholesterol diet (HFD).^44^ Here, NOX1‐derived ROS enhanced peroxynitrite‐induced hepatocellular injury and impaired hepatic microcirculation by reducing the bioavailability of the vasodilator nitric oxide (NO), which accelerated hepatocyte cell death and NASH progression in HFD‐fed mice.^44^

### 2.2 Setanaxib in liver fibrosis: pre‐clinical evidence

Setanaxib has been shown to replicate the hepatoprotective effects seen in multiple NOX1/4 KO models of liver fibrosis. In mice with the superoxide dismutase 1 G37R mutation (SOD1mu), which enhances NOX‐mediated ROS production, setanaxib suppressed liver fibrosis in CCl_4_‐ and BDL‐treated SOD1mu mice.^13^ This was reflected by reduced hepatic collagen deposition and alpha‐smooth muscle actin (α‐SMA) expression to a similar extent to those in WT mice.^13^ Macrophage infiltration and activation, and TNF‐α mRNA expression, were also lowered in CCl_4_‐treated SOD1mu and WT mice following setanaxib treatment, indicating the suppression of liver inflammation in these mice.^13^ Consistent with these data, pre‐treatment of primary culture‐activated HSCs from SOD1mu mice with setanaxib reduced the expression of profibrotic genes, including collagen α1(I), and suppressed ROS production to similar levels as setanaxib‐treated WT HSCs.^13^

In line with these observations, ROS production and HSC activation were reduced in primary HSCs isolated from BDL‐treated mice following pre‐treatment with setanaxib, as shown by the suppression of procollagen α1(I), α‐SMA and TGF‐β expression.^16^ Observations of reduced oxidative stress, hepatocyte apoptosis and liver fibrosis were seen in setanaxib‐treated mice following BDL compared to those treated with solvent.^16^ Similar results were demonstrated in NASH mice, in which markers of inflammation (TNF‐α) and fibrosis (procollagen α1(I), α‐SMA and TGF‐β) were reduced following setanaxib treatment compared with vehicle‐treated mice.^14^

Platelet‐derived growth factor (PDGF) and lipopolysaccharide (LPS) activate profibrotic signalling pathways in HSCs to trigger liver fibrosis.^17^ In CCl_4_‐treated mice, setanaxib lowered PDGF‐induced expression of proliferative genes in HSCs compared with vehicle‐treated HSCs.^17^ Furthermore, decreased production of ROS and proinflammatory chemokines were observed in LPS‐stimulated HSCs, reflecting a reduction in HSC activation.^17^ Similarly, in BDL‐ and CCl_4_‐treated multidrug resistance gene 2 KO mice (*Mdr2* ^−/−^; genetic model of chronic cholestatic liver injury), activation of HSCs and portal fibroblasts was significantly lower in *Mdr2* ^−/−^ mice treated with setanaxib compared with vehicle‐treated *Mdr2* ^−/−^ mice, indicating that setanaxib inhibited cholestatic fibrosis progression.^18^ Furthermore, using partial portal vein ligation (PPVL) to model portal hypertension (PHT) in rats, treatment with setanaxib decreased markers of PHT and mesenteric angiogenesis and reduced ROS production in the mesenteric arteries of PPVL rats compared with vehicle‐treated rats.^15^ PHT is caused by increased intrahepatic resistance to portal blood flow in chronic liver diseases, most commonly cirrhosis, which in turn is caused by ROS‐induced liver fibrosis and the concurrent formation of hyperdynamic circulation.^45^, ^46^ Hyperdynamic circulation can also be induced by various vascular mediators, including NO, whose synthesis and release is driven by endothelial nitric oxide synthase (eNOS), and vascular endothelial growth factor (VEGF).^15^ Importantly, treatment with setanaxib has been shown to decrease H_2_O_2_‐induced mesenteric VEGF expression in PHT rats induced with PPVL, alongside reduced eNOS phosphorylation and NO production in mesenteric arteries compared with vehicle‐treated rats.^15^

### 2.3 Clinical translatability

Pre‐clinical studies demonstrate that the effects of genetic NOX1/4 deficiency are consistent with those of setanaxib in human liver cells and animal models of liver fibrosis.

In liver biopsy samples from patients with stage 2/3 autoimmune hepatitis, NOX4 expression levels were higher in both myofibroblasts and hepatocytes compared with these cells from control patients.^16^ Consistent with these observations, NOX4 mRNA expression was significantly upregulated in liver biopsy samples from NASH patients compared with patients with simple steatosis and healthy controls, with strong signals in hepatocytes.^14^ Likewise, NOX1 and NOX4 expression was increased in the livers of cirrhotic patients compared with the livers of control patients.^17^

Given that setanaxib has been shown to replicate the protective effects of NOX1/4 KO models seen in multiple lines of pre‐clinical evidence, and that NOX1/4 become upregulated in liver biopsies reflective of several human chronic liver diseases, the evidence suggests that the benefits elicited by setanaxib in pre‐clinical models could potentially be translated to human patients, warranting further exploration in a clinical trial setting.

## 3 KIDNEY FIBROSIS

### 3.1 Role of NOX1/4 in kidney fibrosis

**FIGURE 3:**
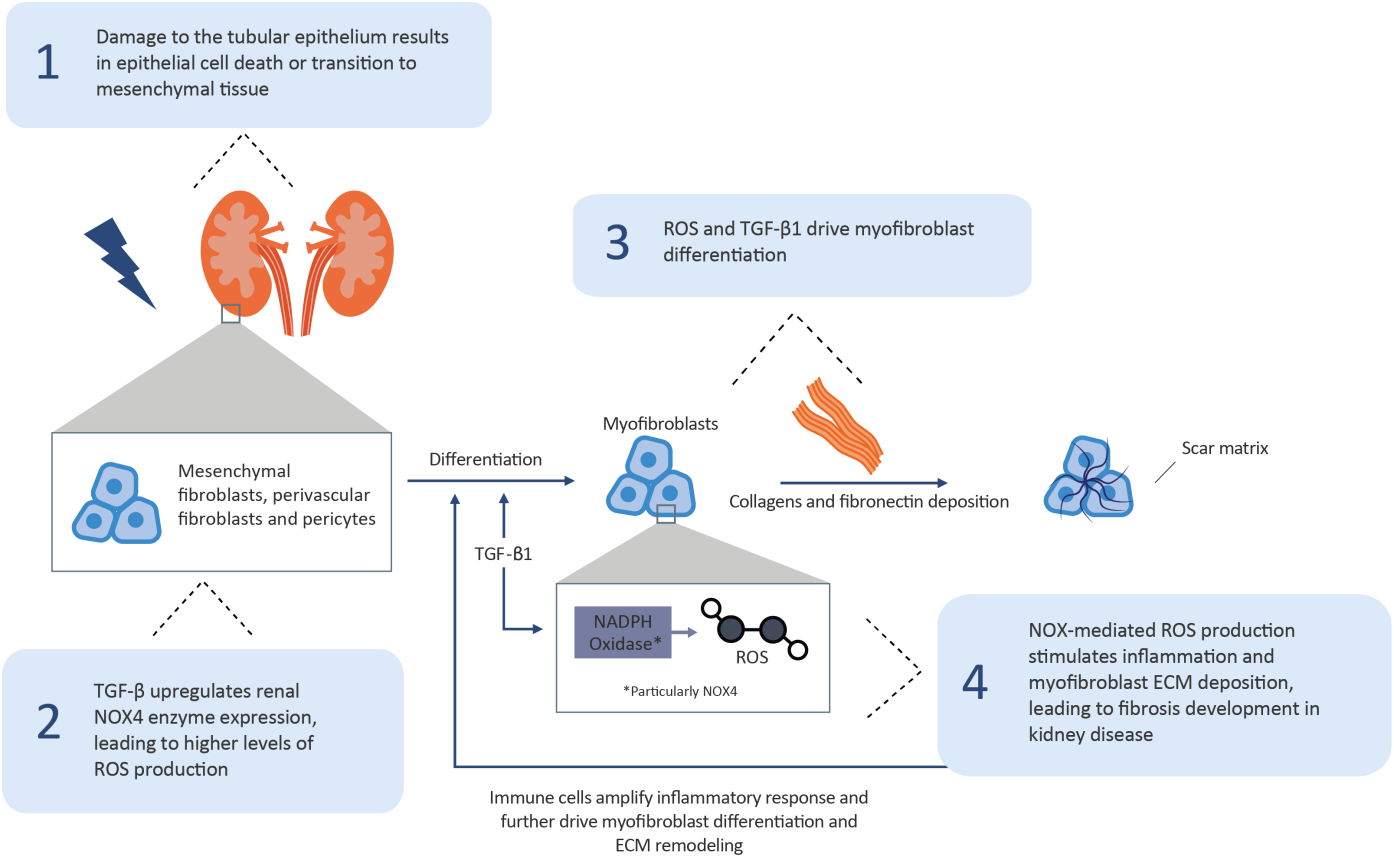
NOX1/4‐mediated ROS production in kidney fibrosis. ECM, extracellular matrix; NOX, NADPH oxidase; ROS, reactive oxygen species; TGF‐β, transforming growth factor‐beta.

Kidney fibrosis is a common pathological consequence of progressive kidney disease that is most commonly caused by diabetes mellitus and hypertension and may ultimately lead to end‐stage kidney disease, regardless of aetiological cause.^10^, ^47^ As with liver fibrosis, myofibroblasts are contractile, activated cells that are responsible for the formation of scar tissue observed in kidney fibrosis.^10^ Damage‐induced tubular epithelial cell death or transition to mesenchymal tissue, termed epithelial–mesenchymal transition (EMT), is thought to initiate myofibroblast formation, along with the differentiation of mesenchymal fibroblasts, pericytes and perivascular fibroblasts into myofibroblasts upon stimulation by profibrotic mediators, such as TGF‐β.^10^ TGF‐β promotes kidney fibrosis by sustaining the EMT of tubular epithelial cells and upregulating NOX‐mediated ROS production, which drives further profibrotic myofibroblast differentiation (Figure 3).^10^

Kidney NOX expression occurs in a cell‐specific manner.^48^ Notably, NOX4 is most abundantly expressed in mesangial cells, podocytes, tubular epithelial cells and endothelial cells.^10^, ^48^ NOX‐derived ROS have been shown to play an important role in many important functional processes within the kidney, such as gluconeogenesis, glucose transport, tubuloglomerular feedback, kidney haemodynamics and electrolyte transport.^10^, ^48^ However, excessive ROS generation evoked by NOXs, most notably NOX4, has been shown to mediate fibrotic pathologies characteristic of many kidney diseases.^11^, ^48^

In a mouse model of streptozotocin‐induced diabetic nephropathy in *ApoE* ^−/−^ mice, the genetic deletion of NOX4 (but not NOX1), protected mice from structural and functional damage linked to diabetic nephropathy.^24^ This was evidenced by the reduction of albuminuria, glomerulosclerosis and mesangial expansion and decreased diabetes‐induced expression of glomerular VEGF, collagen IV and fibronectin in diabetic *NOX4* ^−/−^ *ApoE* ^−/−^ mice versus diabetic *NOX4* ^+/+^ *ApoE* ^−/−^ mice.^24^ Furthermore, podocyte‐specific deletion of NOX4 in streptozotocin‐induced diabetic mice was also linked to reduced albuminuria and glomerular ECM accumulation compared with control mice.^49^ This emphasizes the importance of NOX4 in podocytes in driving fibrosis progression in models of diabetic nephropathy.^49^

Furthermore, deletion of NOX4 in human proximal tubular epithelial cells (HK‐2 cells) suppressed ROS production, proinflammatory marker expression and cellular apoptosis in models of hypoxia‐, colistin‐ and contrast‐induced acute kidney injury (AKI) in vitro.^19^, ^22^, ^23^ Interestingly, however, in a proximal tubular‐specific NOX4 KO mouse model of diabetic kidney disease (DKD), deletion of NOX4 had no beneficial effect on albuminuria, kidney fibrosis or glomerulosclerosis, suggesting that NOX4 localization within the tubular proximal compartment is not essential for DKD progression.^50^ Thus, while there is evidence that NOX4‐derived ROS have an important role in promoting the development of kidney fibrosis, the cell‐type‐related functional differences of NOX4 in fibrotic pathologies must be considered. Notably, while NOX4‐derived ROS trigger inflammation and apoptotic cell death in tubular epithelial cells, they are also important mediators of the activation, migration and transdifferentiation of mesenchymal fibroblasts to profibrotic myofibroblasts, thus contributing to fibrosis development via both direct and indirect mechanisms.^19^, ^22^, ^23^, ^51^

### 3.2 Setanaxib in kidney fibrosis: pre‐clinical evidence

Setanaxib has been shown to replicate the renoprotective effects seen in several NOX4 KO models of kidney fibrosis. In an established murine model of diabetic nephropathy (*ApoE* ^−/−^ mice), setanaxib protected against the development of albuminuria, glomerulosclerosis and mesangial expansion, while significantly decreasing the diabetes‐induced expression of glomerular VEGF, collagen IV and fibronectin in *ApoE* ^−/−^ mice compared with non‐treated *ApoE* ^−/−^ mice.^21^, ^24^ These renoprotective effects occurred alongside the significant reduction of ROS generation in setanaxib‐treated *ApoE* ^−/−^ mice versus untreated *ApoE* ^−/−^ mice.^24^ Additionally, setanaxib significantly lowered markers of fibrosis and inflammation, including TGF‐β and TNF‐α, respectively, to levels comparable to those of untreated non‐diabetic *ApoE* ^−/−^ mice.^21^

Consistent with these data, treatment with setanaxib significantly decreased albuminuria, glomerular hypertrophy and mesangial matrix accumulation to control levels in a mouse model of DKD.^25^ Fumarate is an intermediary molecule in the tricarboxylic acid cycle that may promote the development of kidney pathology by upregulating the expression of profibrotic mediators, such as TGF‐β and hypoxia‐inducible factor 1‐alpha (HIF‐1α).^25^ Fumarate hydratase (FH), a degrader of fumarate, was downregulated in non‐treated diabetic mice alongside the upregulation of TGF‐β and HIF‐1α glomerular expression.^25^ Importantly, treatment with setanaxib restored FH formation and decreased TGF‐β and HIF‐1α expression, which was observed in parallel with the lowering of collagen IV and fibronectin expression to control levels in diabetic mice.^25^

In a mouse model of type 1 diabetes, setanaxib reduced collagen IV and fibronectin expression in cortical, glomerular and tubulointerstitial compartments, which occurred alongside a decrease in glomerular hypertrophy and mesangial matrix accumulation.^20^ These observations were accompanied by the suppression of cortical ROS production, albuminuria, podocyte loss and glomerular macrophage infiltration following setanaxib treatment in diabetic mice, thus protecting against the progression of diabetic nephropathy in type 1 diabetes.^20^

### 3.3 Clinical translatability

Pre‐clinical studies demonstrate that the effects of genetic NOX1/4 deficiency are consistent with those of setanaxib in human kidney cells and animal models of kidney fibrosis.

Incubation of human podocytes in a hyperglycaemic medium caused an increase in NOX4 mRNA expression levels, which was amplified by the addition of TGF‐β.^24^ These observations occurred alongside an increase in ROS generation, and heightened expression of collagen IV, fibronectin and α‐SMA in human podocytes.^24^ Importantly, pre‐treatment of human podocytes with setanaxib decreased the production of ROS and downregulated the expression of these profibrotic markers that are linked to diabetic nephropathy.^24^

In a model of DKD, pre‐treatment with setanaxib significantly decreased H_2_O_2_ production in human embryonic kidney (HEK)‐293 cells transfected with the human influenza haemagglutinin (HA)‐tagged human NOX4 transgene.^25^ Setanaxib has also been shown to replicate the renoprotective effects seen in NOX4 KO models of hypoxia‐, colistin‐ and contrast‐induced AKI, as evidenced by the suppression of ROS production and tubular cell apoptosis in HK‐2 cells.^19^, ^22^, ^23^

Thus, given that the effects of setanaxib in human cell lines replicate those seen in NOX4 KO models, these data suggest that benefits elicited by setanaxib in pre‐clinical models could potentially be translated to human patients, warranting further exploration in a clinical trial setting.

## 4 LUNG FIBROSIS

### 4.1 Role of NOX1/4 in lung fibrosis

Lung fibrosis is characterized by chronic injury repair and ECM deposition in the interstitial‐alveolar spaces and is associated with many of the interstitial lung diseases.^52^, ^53^ Excessive accumulation of fibrotic tissue can lead to reduced lung compliance and increased respiratory effort, as well as hypoxaemia and pulmonary hypertension caused by the collapse of alveolar structural and functional integrity. Together, these factors manifest clinically as the progressive deterioration of respiratory mechanics and gas exchange, which may eventually progress to respiratory failure and death.^52^, ^54^

**FIGURE 4:**
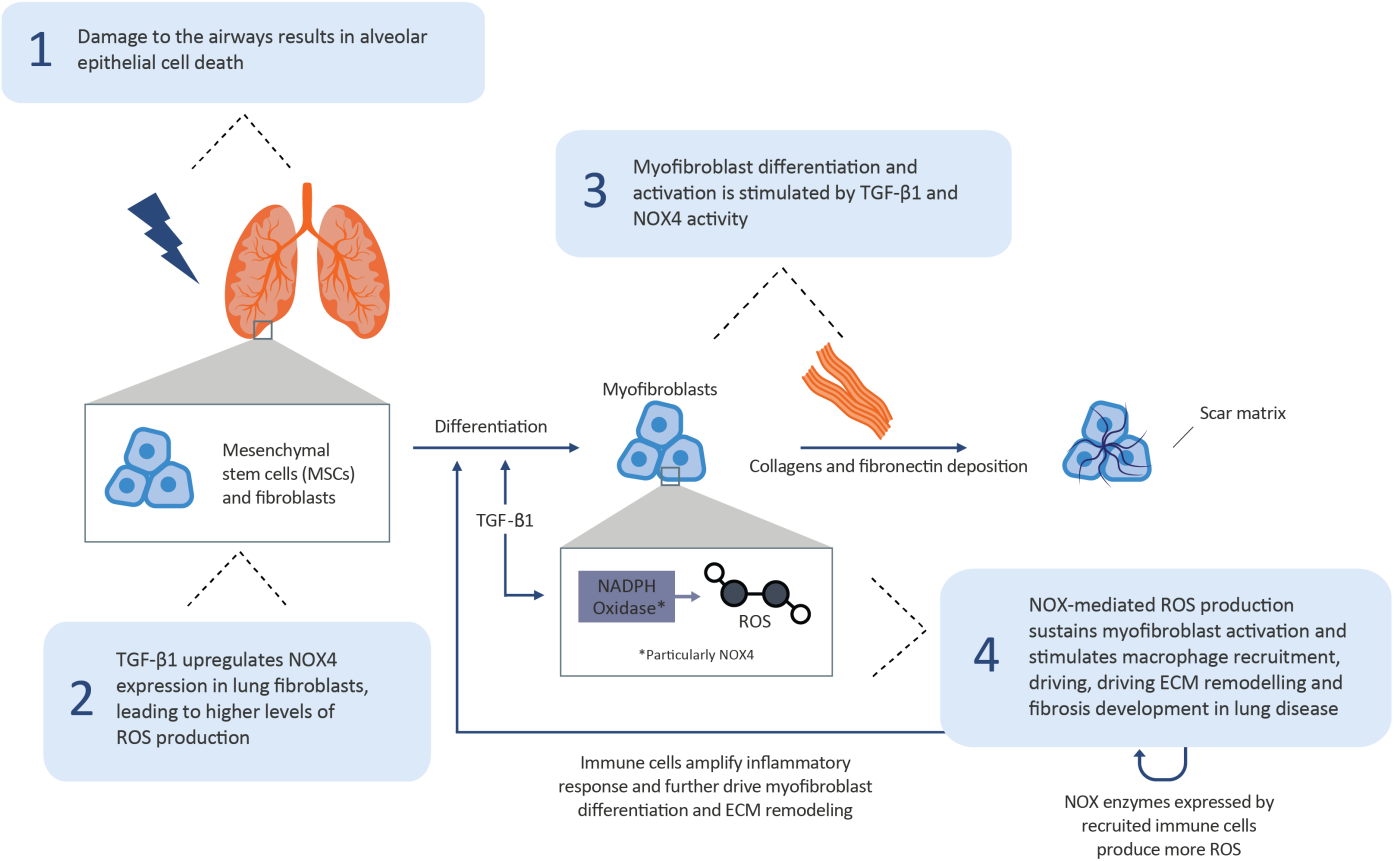
NOX1/4‐mediated ROS production in lung fibrosis. ECM, extracellular matrix; MSC, mesenchymal stem cell; NADPH, nicotinamide adenine dinucleotide phosphate; NOX, NADPH oxidase; ROS, reactive oxygen species; TGF‐β, transforming growth factor‐beta.

Lung fibrosis has diverse aetiologies including drugs, chemical insults, radiation, occupational exposures and connective tissue diseases; alternatively, it may be ‘idiopathic’ in nature.^55^ Damage‐induced alveolar epithelial cell death causes the release of profibrotic mediators, including TGF‐β, which activate resident lung fibroblasts and trigger their differentiation into myofibroblasts, resulting in heightened ECM synthetic capacity and resistance to apoptosis.^10^, ^53^, ^54^ Importantly, TGF‐β induces NOX4‐mediated ROS production in lung fibroblasts, which sustains myofibroblast differentiation and drives fibrosis progression in lung disease (Figure 4).^10^, ^56^ Injured alveolar epithelial cells also release proinflammatory mediators, which trigger the infiltration of activated immune cells to the site of injury.^10^, ^54^ Immune cells in turn amplify inflammatory signalling and contribute to fibrosis development by exacerbating myofibroblast differentiation and ECM deposition.^10^, ^54^ Cellular senescence is also thought to contribute to the pathology of lung fibrosis, in which senescent cells secrete senescence‐associated secretory phenotype factors to promote inflammation, tissue remodelling and cell growth.^10^, ^56^, ^57^

NOX1 is expressed in a range of pulmonary cell types, including pulmonary epithelial cells, pulmonary vascular smooth muscle cells and bronchial epithelial cells.^58^ NOX4 is expressed in macrophages, smooth muscle cells, endothelial cells, mesenchymal cells and epithelial cells.^59^, ^60^, ^61^ Importantly, NOX4 has been implicated in the pathology of lung fibrosis, as evidenced by the reduction of fibroblast senescence and restoration of fibrosis resolution capacity following the siRNA‐induced silencing of NOX4 in bleomycin‐challenged mice with age‐associated pulmonary fibrosis.^28^ Furthermore, NOX4 expression was upregulated following TGF‐β treatment in primary lung fibroblasts isolated from IPF patients, which occurred alongside an increase in NOX4‐mediated H_2_O_2_ generation.^29^

However, while there is evidence demonstrating that NOX1/4 play an important role in progressing lung fibrosis, the cell‐type‐related functional differences of NOX1/4 in fibrotic pathologies must be considered. For example, NOX4 induces the activation and transdifferentiation of pulmonary fibroblasts to myofibroblasts, but triggers apoptosis in pulmonary epithelial cells.^59^, ^62^

### 4.2 Setanaxib in lung fibrosis: pre‐clinical evidence

Setanaxib has been shown to replicate the protective effects seen in NOX4 KO models of lung fibrosis. In C57BL/6J mice with hypoxia‐induced PHT, treatment with setanaxib decreased right ventricular hypertrophy, pulmonary vascular remodelling and proliferation, and reduced TGF‐β expression in the mouse lung.^27^ Similarly, treatment of C57BL/6J mice with setanaxib decreased fibrosis‐induced lung ischaemia reperfusion injury (LIRI), as evidenced by reduced alveolar congestion, alveolar wall thickness, macrophage infiltration and pulmonary cellular apoptosis.^26^

Furthermore, in C57BL/6J mice subjected to bleomycin‐induced lung fibrosis, setanaxib treatment reduced myofibroblast accumulation, as evidenced by lowered α‐SMA expression, and decreased fibroblast senescence compared with vehicle‐treated mice.^28^ This occurred alongside the reversal of age‐associated persistent fibrosis and improved survival, reflected by significantly lower mortality rates in setanaxib‐treated mice compared with vehicle‐treated mice.^28^ Similarly, ex vivo treatment of IPF lung fibroblasts isolated from C57BL/6 J mice with setanaxib lowered H_2_O_2_ production and cellular senescence in IPF fibroblasts, as evidenced by a reduction in senescence‐associated beta‐galactosidase activity compared with vehicle‐treated fibroblasts. Additionally, setanaxib improved the susceptibility of fibroblasts to apoptosis, reflected by the increased caspase‐3 activity.^28^

### 4.3 Clinical translatability

Several lines of pre‐clinical evidence have demonstrated that effects of genetic NOX1/4 deficiency are consistent with those of setanaxib in human lung cells and animal models of lung fibrosis.^63^

In hypoxia‐exposed human pulmonary artery endothelial and smooth muscle cells (HPAECs and HPASMCs), treatment with setanaxib reduced hypoxia‐induced proliferation of HPAECs and HPASMCs, reflecting a decrease in pulmonary vascular cell proliferation.^27^ Additionally, setanaxib lowered hypoxia‐induced H_2_O_2_ generation in HPAECs and HPASMCs, and protected against hypoxia‐associated increases in TGF‐β expression and decreases in peroxisome proliferator‐activated receptor gamma expression in these cells.^27^ Consistent with these findings, treatment of human lung fibroblasts with setanaxib reduced TGF‐β‐induced H_2_O_2_ generation, along with lowering fibroblast differentiation and TGF‐β‐induced expression of fibronectin and α‐SMA.^29^

Given that NOX4 expression is upregulated in lung fibroblasts isolated from IPF patients, and that the protective effects of setanaxib in human cell lines replicate those seen in NOX4 KO models, these data strengthen evidence suggesting that the benefits of setanaxib treatment seen in pre‐clinical models could potentially be translated to human patients, warranting further exploration in a clinical trial setting.

## 5 PERSPECTIVES AND FUTURE DIRECTIONS

While there is a wealth of pre‐clinical evidence that supports the protective role of NOX1/4 inhibition by setanaxib in fibrotic pathologies implicated in liver, kidney and lung disease, the mechanisms underlying NOX1/4‐induced fibrosis and the selective inhibitory action of setanaxib require further investigation.

Although several studies have demonstrated that the genetic deletion or pharmacological inhibition of NOX1/4 confers protective effects against fibrosis, conflicting evidence exists suggesting that NOX1/4 deficiency may promote fibrosis.^64^, ^65^ This was shown in mice subjected to unilateral ureteral obstruction (UUO), a model of tubular stress in the kidneys leading to kidney fibrosis in chronic kidney disease (CKD), where tubulointerstitial fibrosis and tubular epithelial cell apoptosis were significantly increased, and peritubular capillary density was significantly decreased, in NOX4 KO mice compared with WT mice.^65^ Importantly, oxidative stress was not reduced but increased in NOX4 KO mice subjected to UUO compared with WT mice, suggesting that NOX4 plays an antioxidant role.^65^ Collectively, these data therefore suggest that NOX4 may protect against kidney fibrosis in CKD by counteracting oxidative stress, lowering apoptosis and maintaining microvascularization in kidney tubular cells.^65^

The selective inhibition of NOX1/4‐driven ROS production by setanaxib is supported by multiple lines of evidence. However, it has been proposed that effects induced by setanaxib are independent of NOX1/4 activity and the compound may instead modulate ROS metabolism through other mechanisms, for example peroxidase inhibition.^66^, ^67^, ^68^ Thus, the pharmacological characterization of setanaxib in terms of its selectivity and mode of action requires further exploration. To clearly distinguish between a true NOX inhibitor and molecules with ROS‐scavenging and/or assay‐interfering properties, a series of biochemical assays have previously been used.^66^, ^67^, ^68^ Here, setanaxib demonstrated substantial interference with peroxidase‐dependent assays and potently inhibited the H_2_O_2_‐producing activity of xanthine oxidase in the absence of NOX enzymes; these effects potentially originated from direct xanthine oxidase inhibition or H_2_O_2_ scavenging, or the non‐specific inhibition of horseradish peroxidase used in the assay.^66^, ^68^ Thus, while setanaxib is the most widely recognized NOX1/4 dual inhibitor, its inhibitory action on ROS production may not fully stem from the inhibition of NOX1/4, but rather from an unspecified redox mechanism that merits further investigation.^66^, ^68^

Therefore, while there have been significant advances in the understanding of the pathophysiology underlying liver, kidney and lung fibrosis, the precise roles played by NOX1/4 in their pathophysiology and the selective inhibitory action of setanaxib require further exploration.

## 6 CONCLUSIONS

NOX1/4‐mediated ROS production is a fundamental driver of injury‐induced fibrotic pathologies common to many chronic inflammatory diseases in animal and cellular pre‐clinical models within the liver, kidney and lung. Dual NOX1/4 inhibition and/or redox modulation by setanaxib has been shown to replicate the protective effects evoked by NOX1/4 deficiency within each of these organ classes. Genetic models of NOX1/4 deficiency largely replicate the effects of setanaxib in human cell lines and animal models of fibrosis, which provides rationale to further explore NOX1/4 inhibition by setanaxib in fibrotic pathologies in human patients enrolled in controlled clinical trials. However, there are still knowledge gaps concerning the precise role of NOX1/4 in fibrotic pathologies that underlie liver, kidney and lung disease, and the selective inhibitory action of setanaxib has not been fully elucidated. Thus, while there is a wealth of evidence in support of setanaxib as a potentially promising therapeutic intervention to attenuate fibrosis in a pre‐clinical setting, the clinical translatability of this evidence requires further exploration. The results from current and future large, phase 2b/3 clinical trials will further deduce the efficacy and safety of setanaxib.

## AUTHOR CONTRIBUTIONS

**Victor J Thannickal:** Conceptualization (equal); formal analysis (equal); investigation (equal); validation (equal); visualization (equal); writing – original draft (equal); writing – review and editing (equal). **Karin Jandeleit‐Dahm:** Conceptualization (equal); formal analysis (equal); investigation (equal); validation (equal); visualization (equal); writing – original draft (equal); writing – review and editing (equal). **Cédric Szyndralewiez:** Conceptualization (equal); formal analysis (equal); investigation (equal); methodology (lead); validation (equal); visualization (equal); writing – original draft (equal); writing – review and editing (equal). **Natalie J. Torok:** Conceptualization (equal); formal analysis (equal); investigation (equal); validation (equal); visualization (equal); writing – original draft (equal); writing – review and editing (equal).

## FUNDING INFORMATION

This study was sponsored by Calliditas Therapeutics AB. Support for third‐party writing assistance for this article was funded by Calliditas Therapeutics AB in accordance with Good Publication Practice (GPP3) guidelines (http://www.ismpp.org/gpp3).

## CONFLICT OF INTEREST

VJT, KJD, NJT: The authors confirm that there are no conflicts of interest. CS: Former employee and shareholder of Calliditas Therapeutics Suisse SA.

## Data Availability

Data sharing is not applicable to this article, as no new data have been generated.
